# The Homœopathic Physician’s Visiting List and Repertory

**Published:** 1888-03

**Authors:** 


THE HOMŒOPATHIC PHYSICIAN'S VISITING LIST AND REPERTORY. By Robert Faulkner, M. D. Bœricke & Tafel, New York and Philadelphia. 1888.

This visiting list is especially adapted for homœopathic physicians. For this purpose the columns are ruled wide, that the name of the remedy may be inserted opposite the name of the patient. Moreover, it is perpetual, being therefore useful for any year or any month. In the forepart is a condensed repertory to the more commonly used homœopathic remedies. This repertory must prove of much service to many a physician in moments of doubt. Finally, there are an ordinary calendar, an obstetric calendar, a table on antidotes to poisons, and rules of procedure in cases of asphyxia. W. M. J.

---

### NOTES AND NOTICES.

ANOTHER.—A subscriber writes: "Let me say that your journal is worth more to a *homœopath* than all our other homœopathic journals combined. I am just re-reading volume seventh. It is as fresh and as new now as it was when I first read it. Yours sincerely, S. W. C."

